# Therapeutic targeting of cancer cell cycle using proteasome inhibitors

**DOI:** 10.1186/1747-1028-7-26

**Published:** 2012-12-26

**Authors:** Namrata Rastogi, Durga Prasad Mishra

**Affiliations:** 1 Cell Death Research Laboratory, Division of Endocrinology, CSIR- Central Drug Research Institute, Lucknow, 226001, India

**Keywords:** Proteasome, Cell division, Proteasome inhibitors, Cell cycle, Cancer

## Abstract

Proteasomes are multicatalytic protease complexes in the cell, involved in the non-lysosomal recycling of intra-cellular proteins. Proteasomes play a critical role in regulation of cell division in both normal as well as cancer cells. In cancer cells this homeostatic function is deregulated leading to the hyperactivation of the proteasomes. Proteasome inhibitors (PIs) are a class of compounds, which either reversibly or irreversibly block the activity of proteasomes and induce cancer cell death. Interference of PIs with the ubiquitin proteasome pathway (UPP) involved in protein turnover in the cell leads to the accumulation of proteins engaged in cell cycle progression, which ultimately put a halt to cancer cell division and induce apoptosis. Upregulation of many tumor suppressor proteins involved in cell cycle arrest are known to play a role in PI induced cell cycle arrest in a variety of cancer cells. Although many PIs target the proteasomes, not all of them are effective in cancer therapy. Some cancers develop resistance against proteasome inhibition by possibly activating compensatory signaling pathways. However, the details of the activation of these pathways and their contribution to resistance to PI therapy remain obscure. Delineation of these pathways may help in checking resistance against PIs and deducing effective combinational approaches for improved treatment strategies. This review will discuss some of the signaling pathways related to proteasome inhibition and cell division that may help explain the basis of resistance of some cancers to proteasome inhibitors and underline the need for usage of PIs in combination with traditional chemotherapy.

## Introduction

Cancer cells differ from the normal cells of the body in their ability to divide indefinitely and evade programmed cell death [1]. In normal cells, the cell cycle is controlled by a complex series of signaling pathways by which a cell grows, replicates its DNA and divides. This process also includes mechanisms which ensure that any errors therein are corrected, and if not, the cells commit suicide in a systematic cellular process known as programmed cell death or apoptosis. In cancer, as a result of genetic mutations, this regulatory process malfunctions, resulting in uncontrolled cell proliferation. Alteration in these mechanisms commonly affect the expression of cell cycle regulatory proteins, causing overexpression of cyclins and loss of expression of cyclin dependent kinase inhibitors (CKIs). Therefore CDK inhibitors and related transcription factors have long been viewed as potential targets for anticancer therapeutics. Despite years of research and attempts directed at inhibiting cell cycle kinases or cell cycle regulating transcription factors, most of these approaches have not been successfully translated to the clinic as cancer therapeutics [2]. Therefore, targeting the pathways which bring about timely degradation of these proteins *i.e.* Proteasome pathway, provided the rationale for targeted anticancer drug development.

Cells normally depend upon two important pathways for degradation of cellular proteins, *i.e.* 1. the aggresome/lysosome pathway for extracellular proteins and 2. the ubiquitin proteasome pathway ( UPP) for intracellular proteins [3]. Proteasomes, key complexes of the latter pathway, are multicatalytic protease complexes engaged in non-lysosomal recycling of intra-cellular proteins of short life span. These small protein degradation machines are present ubiquitously in both cytoplasm and nucleus of mammalian cells. These are constituted of a ATP-dependent 26S core complex consisting of 20S catalytic core capped by a 19S regulatory subunit at both ends [3]. They guide the proteolytic cleavage and recycling of proteins whose functions are needed to be checked on a timely and stage specific manner [4-7]. Target proteins of the UPP consist of a polyubiquitin chain covalently attached to their lysine residues. This ubiquitin tag is recognized by the 19S regulatory subunit of the proteasome and the tagged protein is degraded in a ATP dependent manner [8]. Apart, from their presence in healthy cells they are found to be highly expressed and active in cancer cells. In cancer cells, proteasomes are engaged in proteolysis of many tumor suppressor proteins related to cell division. Owing to their established role in cancer progression, several compounds with proteasome inhibition activity has been tested and recognized as potential anti-cancer drugs against hematological malignancies and some solid cancers [9-12]. Based on their mode of action, compounds having proteasome inhibitory activity have either reversible or irreversible mechanisms of action [13]. Proteasome inhibitors can be of two types depending upon the source of origin *i.e.* synthetic and natural. Bortezomib ( Valcade, PS-341), a dipeptide boronic acid, first of its class of compounds, has been well studied for its proteasome inhibitory activity [14].Bortezomib was discovered to be a synthetic proteasome inhibitor with reversible mechanism of action [15]. Long tested for its anti cancer properties in multiple myeloma, it was approved by FDA for treatment of patients with multiple myeloma [16,17]. Bortezomib has also been shown to exert anti-cancer activity against many cancer types [18,19]. More recently compounds with irreversible mode of action are considered to be the second generation of proteasome inhibitors and have shown promising results in clinical trials namely Carfilzomib (PR-171), Salinosporamide A (NP-0052) and MLN9708 [20,21]. Moreover many such compounds are also being tested currently in pre-clinical studies on multiple cancer cell lines [9,22-26] (Table 1). Inhibition of proteasomal activity targets the cancer cell in a multipronged manner including, inhibition of proliferation and induction of cell cycle arrest, induction autophagy and apoptosis. As uncontrolled cell division is the root cause of any cell acquiring malignancy, cell cycle and cell division are popular targets for limiting cancer cell proliferation. Most of the PIs studied till date arrest cancer cells in different phases of cell cycle thereby deregulating cancer cell division (Table 2). Some cancer cells often develop resistance to proteasome inhibition thereby limiting their use in cancer therapy. However the details of the related signaling pathways and molecular mechanisms activated in response to proteasome inhibition require detailed investigation. This review will briefly discuss some of these signaling pathways / mechanisms regulated by PIs and their possible role in influencing resistance to proteasome inhibition therapy.

**Table 1:** List of proteasome inhibitors natural and synthetic in pre-clinical studies and clinical trials

FDA approved^*^/ In Clinical trials		**In preclinical Studies**			
**Synthetic**	**Natural**	**Synthetic**	**Natural**		
Bortezomib^*^[9]	NPI-0052 [13]	CEP-18770 [12]	Celastrol [14]
Carfilzomib (PR-171) [16]			Shikonin [16]
MLN9708 [16]			EGCG [18]
			Curcumin [19]

**Table 2:** Table showing relationship between cell cycle arrests with some of the known proteasome inhibitors in different cancer types

**Proteasome Inhibitor**	**Cancer Type**	**Cell cycle phase arrest**	**Proteins/ Pathways involved**
**Bortezomib**	Colorectal caner [8]	G2/M	UBE2C, cyclin A and cyclin B1
	Malignant pleural mesothelioma (MPM), Breast cancer [51]	G2/M	p21
	CML [42]	G2/M	Rb, NF-ĸB
	Glioblatoma multiforme (GBM) [28]	G2/M	JNK, Cyclin B, p21, p27, CDK2, CDK4 and E2F4
**MG-132**	Lung cancer (Calu 6) [46]	S	ROS generation and GSH depletion
	Cervical cancer [55]	G2/M	ROS generation and GSH depletion
	Gastric cancer [47]	G2/M	Macroautophagy
**Sanggenon C**	Leukemia [13]	G0/G1	p27
**Isothiocyanates**	Multiple myeloma [12]	G2/M	p53, IĸB, ROS generation
**BSc2118**	Multiple myeloma [11]	G2/M	p21, NF-ĸB
**Celastrol**	Cervical cancer [62]	G2/M	Autophagy and paraptosis

### Proteasome inhibitors and cancer cell division

#### Proteasomes are critical for maintenance of cancer cell division

Cell division in cells is the interplay of several cell cycle regulatory proteins with restricted life span. Major proteins related to cell cycle process are cyclins, cyclin dependent kinases (CDKs), CDK inhibitors (CKIs) and some transcription factors. Among these cyclins/CDK complexes remains to be the central players in ensuring rapid cell division by continuously reshuffling their core partners for subsequent cell cycle phase transitions. Each complex type is assigned a role in a particular cell cycle phase and every time the replaced partner is degraded to mark the onset of the next phase. Cyclin proteins are found to be highly upregulated in cases of aberrant cell division in cancer cells. Particularly cyclins D and E are frequently upregulated in different cancer types [27,28]. This upregulation of cyclins is further supported by the down regulation of another class of CDK regulatory proteins, the CKIs, which bind, inactivate and degrade the cyclin/CDK complex. Rapid proteasomal degradation of CKIs in subsequent cell cycle phases contribute to the uncontrolled cell division in cancer cells. Recent studies have established the involvement of ubiquitin proteasome pathway (UPP) in degradation and recycling of the above mentioned class of proteins, crucial for cancer onset and progression [29]. The proteasomal degradation of proteins is well complemented by the ubiquitin conjugating enzymes E1, E2 and E3 ligases, which add multiple ubiquitin molecules to the proteasome substrate proteins. Mdm2, Skp 1/Cul 1/F-box protein (SCF), Anaphase promoting complex (APC), Ubiquitin-conjugating enzyme E2C (UBE2C) are among the busiest ligases involved in cell cycle regulation. Most of these enzymes target the proteolysis of tumor suppressor proteins like p53, p21 and p27. The expression and activity of some of these enzymes has also been reported to be altered by PIs in many cancer cells [29]. Proteasomes are also involved in the regulation of cell division in response to DNA damage response (DDR) signals mediating F-box protein triggered degradation of cyclin D1 protein and G1 cell cycle arrest [30].

#### PIs disrupt cancer cell division

Proteasome inhibitors inhibit the activity of the proteasome complex and in turn attenuate its protein degradation activity. Therefore, PIs lead to the accumulation of those proteins which are marked for ubiquitin-proteasome mediated degradation in cancer cells. These proteins includes CKIs, various transcription factors, DNA repair enzymes, kinases and cyclin dependent kinase inhibitors (CKIs). Generally, the prime function of CKIs lies in degrading cyclin/CDK complexes and disrupting cell division. CKIs, p21 and p27 are known to be suppressed in several cancer types and are attributed to cancer progression [31,32]. Bortezomib, along with other PIs has been reported to significantly increase the expression of p21 and p27 proteins in many cancers thereby causing cell cycle arrest [33-35]. On the other side proteasome inhibition also cause accumulation of the tumor suppressor p53 which is a crucial component of cell cycle regulation. PIs abrogate the degradation of p53 in cancer cell reactivating its function in G1/S and G2/M arrest [29]. Another key component of PI mediated growth arrest and apoptosis is inhibition of NFkB signaling and its downstream target proteins mainly cyclin D, responsible for G1/S transition and commitment to DNA synthesis [36]. Accumulation of non degraded proteins increase unfolded protein response (UPR) leading to induction of endoplasmic reticulum stress which again causes cell cycle arrest to release cells from this cellular stress. PIs induced ROS generation in many cancers is responsible for DNA damage mediated cell cycle arrest wherein cells rigorously perform DNA repair mechanisms [36]. p53 along with some other kinases either help in this process through DNA repair enzymes or alternatively induce apoptosis in case of serious genotoxic stress [37]. PIs have also been reported to modulate the activity of MAPK pathways of cell proliferation [36].Relationship between these signaling pathways and proteasome inhibitors in cancer cells are discussed in the subsequent portions of this review.

### PI induced signaling pathways

#### NFkB signaling pathway: a favorite choice

**Figure 1:**
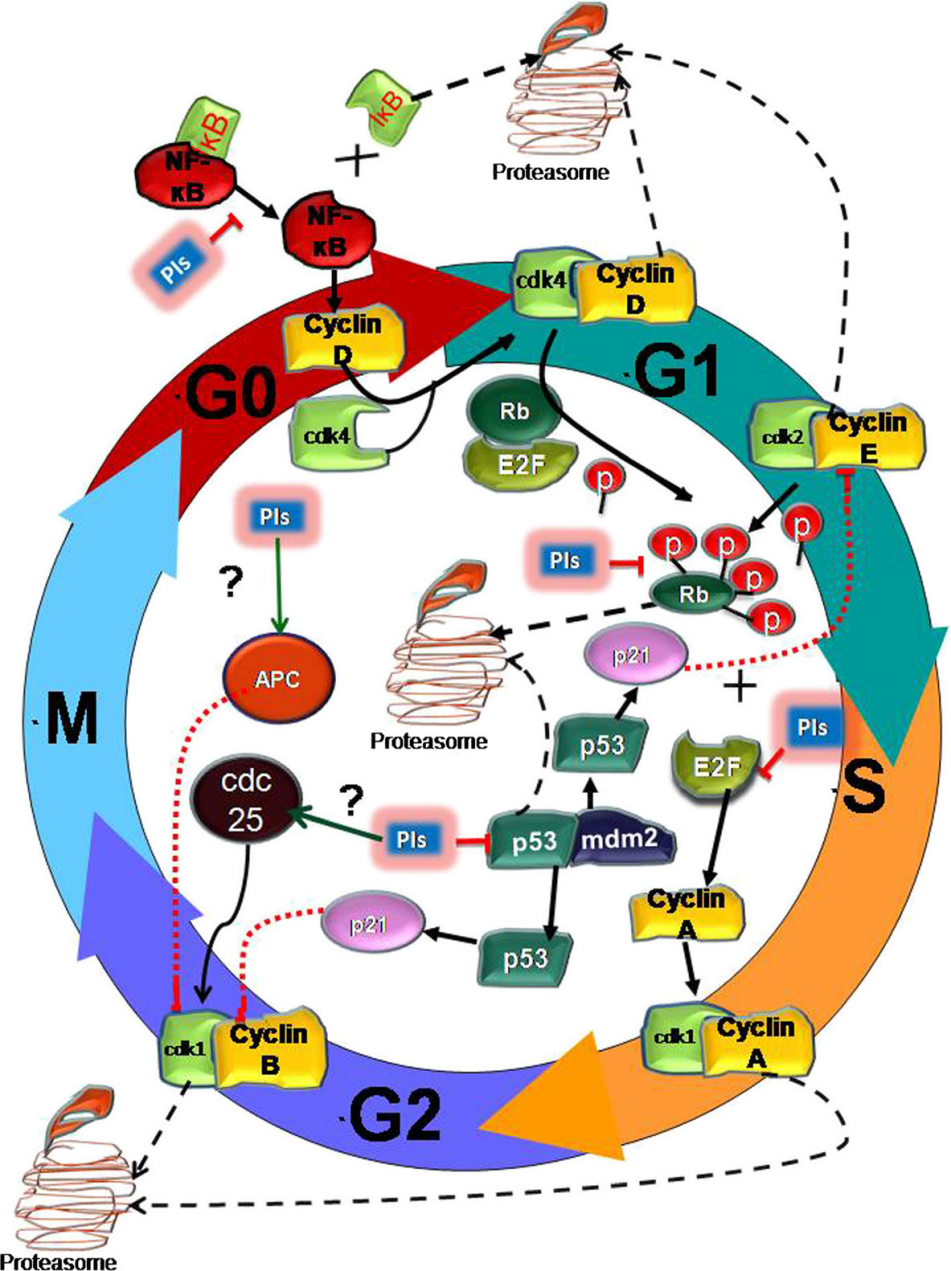
**Regulation of NFkB, E2F/Rb and p53/p21 pathways of cell cycle arrest by PIs.** Proteasome inhibitors block the degradation of IĸB, an inhibitor of NFĸB via proteasome. This causes the accumulation of NF-ĸB in cytosol and represses its transcriptional activity which further inhibits CyclinD1 expression. Decreased Cyclin D1 level terminates its binding to CDK4, which in turn fails to hypo-phosphorylate the Rb protein. Once hypo-phosphorylation of Rb protein is ceased its hyper-phosphorylation by Cyclin E/ CDK2 complex is abrogated and its dissociation from E2F and proteasomal degradation is also inhibited. Undissociated E2F fails to express CyclinA genes and genes involved in DNA synthesis and S phase progression. On the other hand, proteasome inhibitors also inhibit mdm2 mediated degradation of p53 protein leading to the reactivation of its transcriptional activity. p53 reactivation increases p21 expression, a CKI, which inhibit cyclin E/ CDK2 complex ceasing G1 phase progression. However p21 also is known to inhibit cyclin B/ CDK1 complex causing G2/M arrest. Proteins like APC and cdc25 family members are also key players in the sG2/M phase of cell cycle but their modulation by PIs still remains unclear.

NFkB is one of the major transcription factors critical for inflammatory cytokine expression in many cancer cells. NFkB signaling in cancer cells is controlled by the ubiquitin proteasome pathway which directs the ubiquitination and proteolysis of its inhibitory partner, IĸB. Once freed from its inhibitor, NFkB gets localized in the nucleus where it allows transcription of many genes driving cancer cell proliferation [38]. NFkB pathway is constitutively activated in cancers and play a crucial role in conferring resistance to radiation and chemotherapeutic agents in cancers [39,40].However, among many proteins activated by NFkB, Cyclin D plays a crucial role in carcinogenesis. Cyclin D binds to CDK 4/6 in the initial phase of G1 to S phase transition where it performs two major functions, hypophosphorylation of retinoblastoma protein (Rb) [41] and protection of cyclin E/ CDK2 complex from inhibitory effect of p21 and p27 [27] (Figure 1).

#### MAPK pathway: modulating cellular division

**Figure 2:**
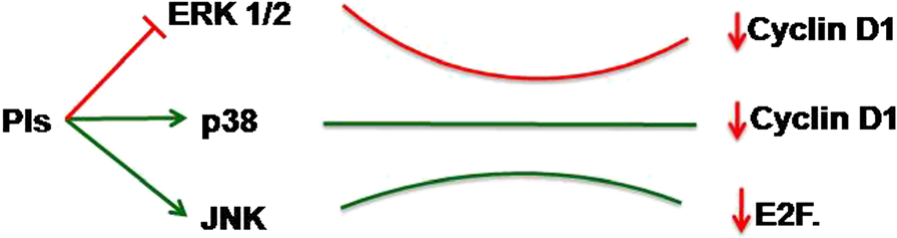
**MAPK pathways and Proteasome inhibitors: Proteasome inhibitors modulate the members of MAPK pathway and control cell cycle of cancer cells.** PIs inhibit the activation of ERK 1/2 protein, which is found to increase the expression of cyclin D1. PIs activate p38 MAPK which antagonizes the effect of ERK 1/2 and decrease cyclin D1. PIs also activate JNK which is known to inhibit the E2F activity.

Mitogen activated protein kinases (MAPKs) family proteins are involved in many processes of tumor development and progression [39]. MAPK are associated with cell survival and apoptosis in stressful cellular environments [39]. Among the family members, ERK1/2 (p44/42) is associated with cell survival and tumor progression where as the other two members, p38 MAPK and c-Jun N-Terminal kinases (JNK) are linked to either cell survival or apoptosis [39]. Though not much has been explored regarding how PIs modulate these pathways but there are evidences which clearly indicated crosstalk between them [39]. According to a previous report addition of PIs decreased the level of phosphorylated ERK1/2 in breast cancer cell lines through activation of MKP-1, a phosphatase, generally degraded by the proteasome pathway [39]. Ras / MEK/ERK pathway regulates the expression of Cyclin D1 by modulating AP-1 and ETS transcription factor, thereby playing a critical role in G1 progression [42-44]. Bortezomib has also been documented to abrogate the activation of p44/42 (ERK1/2) protein along with PI3K/Akt/mTOR and inhibition of the nuclear export of HIF-1α, however its relation to cell cycle arrest remains elusive [45]. Inactivation of ERK by PIs may lead to cell cycle arrest in cancer cells. Secondly, p38 MAP kinases signaling also modulate mammalian cell cycle by antagonizing the effects of ERK mediated Cyclin D expression and thus regulating the G1/ S cell cycle arrest. MG-132 treatment significantly increased the phosphorylation of p38 MAPK in Calu-6 cells and its inhibition partially inhibited MG-132 induced cell death [46]. Lastly, c-Jun-N-terminal Kinase, the third component of the MAPK pathway, has been reported to be activated in MG-132 induced apoptosis [47]. Bortezomib treatment activated JNK in glioblastma multiforme (GBM) cells causing G2/M arrest with subsequent upregulation of p21 and p27 proteins and simultaneous decrease in cyclin B, CDK2, CDK4 and E2F4 protein levels [48]. More recently Bortezomib has also been reported to activate JNK in head and neck cancer cells [49]. JNK1 binds and phosphorylates E2F and limit its ability to bind to DNA, this may induce G1 arrest subsequent to CDK mediated phosphorylation of pRb [50]. On the other hand its activation leads to cell proliferation by increasing the expression of cyclin D1 and repression of p53 and p21 proteins [51,52]. However correlation of JNK to the cell cycle arrest is still a matter of investigation. Involvement of MAPK pathways in cell cycle and PI induced cell death pave the way for identification of many of the novel targets which are supposed to provide a mechanistic link between these two processes (Figure 2).

#### p53/21 pathway: blocking the doorstep of cell cycle entry

Transcription factor p53 lies in very close association with both proteasome and cell cycle pathways [53]. It has been the most extensively studied protein of its class in relation to PIs. In cancer cells, p53 protein is marked for proteasomal degradation by mdm2 protein, a E3 type of ubiquitin ligase [53-55]. Mdm2 ligase is found to be highly expressed in cancers, though the underlying mechanism is still unclear, possibly to keep p53 protein away from tumor formation processes [56]. Proteasomal inhibition allows accumulation of p53 and its nuclear export in cancer cells and thereby increase the expression of its transcriptional target gene p21. p21, a potent CDK inhibitor binds and inactivate cyclin E and the CDK2 complex. This complex is essential for late G1 phase of cell cycle mediating entry in the S phase of cell cycle (Figure 1). However activation of p53 and p21 proteins leads to G0/G1 cell cycle arrest but probably there are several other mediators of this pathway regulating PI induced G0/G1 arrest.

#### Rb/E2F pathway: regulating DNA synthesis

Rapid proteasomal degradation of the retinoblastoma protein is evident in several cancers. Many oncoproteins are engaged in triggering ubiquitin proteasome degradation of Rb including viral oncoprotein E7 of human pappiloma virus type 16, Epstein-Barr virus nuclear antigen 3C (EBNA3C) and gankyrin [57-62]. Rb act as a tumor suppressor protein playing crucial role in cell cycle regulation, DNA replication, DNA damage repair and many other cellular processes. The preliminary role of Rb in cell cycle regulation is binding and stabilization of E2F family of proteins causing their transcriptional repression. Transcriptionally repressed E2F becomes ineffective in activating genes required for DNA synthesis and S phase progression [63]. Administration of PIs mediates Rb protein escape from proteasomal degradation. Non-degraded Rb still remains bounded to E2F which indirectly inhibits E2F mediated expression of S phase progression genes thereby arresting cancer cells in G0/G1 or S phase (Figure 1). According to a recent study , Bortezomib induced cell cycle arrest and apoptosis in BCR/Abl expressing, imatinib resistant and sensitive CML cells. The molecular mechanism involved inhibition of both NFĸB and phosphorylation of Rb eventually leading to caspase dependent apoptosis [64]. However, more elaborate mechanism of cell cycle regulation by Rb/E2F pathway is still not understood with respect to the proteasome inhibitors.

#### DNA damage check point pathway: enforcing G2/M phase arrest

**Figure 3:**
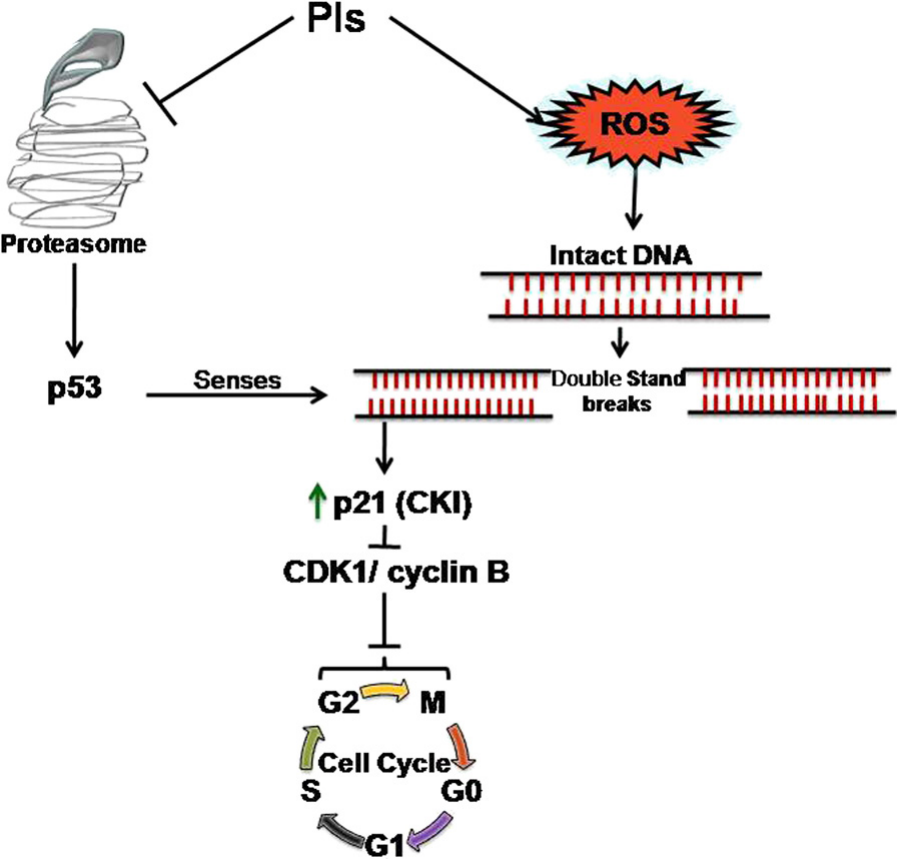
**Proteasome inhibitor induced ROS generation and cell cycle arrest.** Proteasome inhibitors increase the endogenous level of reactive oxygen species which causes DNA damage by inducing double strand breaks in intact DNA. PIs treatment leads to accumulation of p53 which senses this DNA damage. p53 reactivation upregulates p21 expression, a CDK inhibitor. p21 binds and inactivates Cyclin B and CDK2 and causes G2/M cell cycle arrest in response to PIs.

Generally DNA damage checkpoint is imposed by formation of double stranded breaks to the intact DNA by any DNA damaging agent or generation of intracellular ROS [65-68]. DNA damage and break in DNA replication process most often cause G2/M or S phase cell cycle arrest [69-72]. Several reports have established that PIs induced G2/M arrest in cancer cells by activation of p53 and p21 proteins [73,74] (Figure 3). A recent study has shown that PSI-341 treatment lead to p53 dependent G2/M arrest upon co-treatment with DNA damaging agent SN-38 [39]. PSI-341 also reduced the expression of two p53 regulated proteins 14-3-3sigma and survivin both of which regulate G2/M progression and apoptosis [75]. p53 dependent G2/M arrest after generation of ROS has also been reported in isothiocyanate treated multiple myeloma cells [34]. MG-132 has been reported to cause generation of ROS and depletion of the antioxidant GSH in Calu-6 lung cancer cells and cervical cancer leading to S and G2/M phase arrest respectively [46,76]. Other signaling proteins viz, ATM, ATR, Chk1, Chk2 kinases and cdc25 family of phosphatases also lie close to the pathway DDR and promote cell cycle arrest both in p53 dependent and independent manner. Recycling of these proteins is performed by the UPP [77,78]. Though their role in PI induced cell cycle arrest is a matter of further research.

#### Endoplasmic reticulum stress pathway inducing autophagy mediating cell death

**Figure 4:**
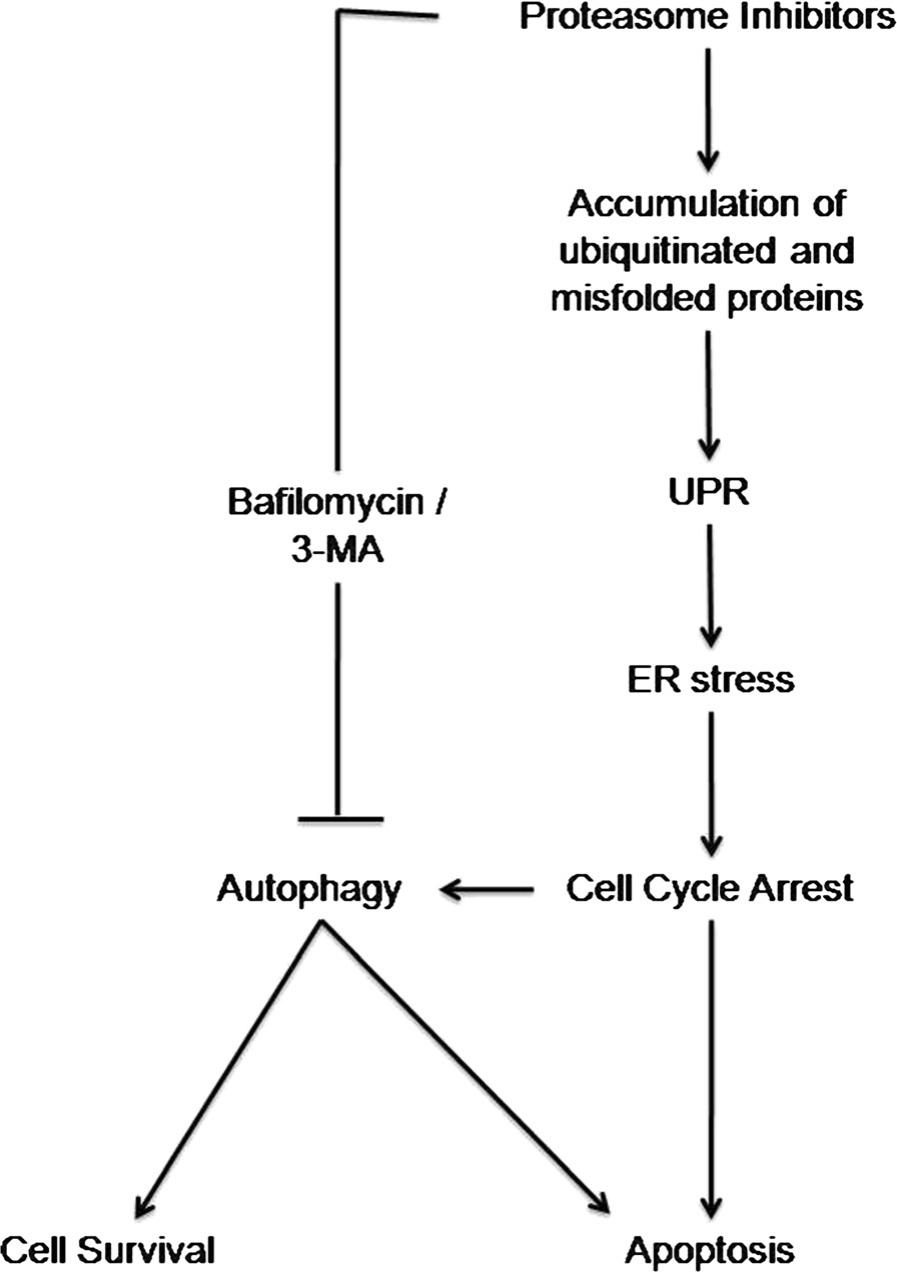
**Association of ER stress pathway and autophagy in Proteasome inhibitor mediated cell cycle arrest.** Proteasome inhibitor treatment arrests the normal functioning of the proteasome and increases the load of ubiquitinated and misfolded proteins in cancer cells. This accumulation generates unfolded protein response (UPR) and consequently ER stress. ER stress generation enforces cells to be arrested in cell cycle phases and induce autophagy in order to compensate and reduce the non-functional protein load. At this stage autophagy can resolve the normal cell state by digesting the non-functional proteins and promote cancer cell survival or can trigger apoptosis leading to cancer cell death. Autophagy inhibitors 3-MA and Bafilomycin A can modulate the effect of autophagy induced cell cycle arrest or apoptosis in association with PIs.

PIs increase the load of misfolded and ubiquitinated proteins in cancer cells which elicit endoplasmic reticulum stress. ER stress induced non-functional protein overload leads to formation of autophagosome to digest these intracellular proteins by a process known as macroautophagy [49]. Macroautophgy induction is thought to overcome the protein load and restore normal cell division [79,80]. Autophagy is the process of degradation of intracellular proteins next to ubiquitin proteasome pathway and possess dual role in PI induced cancer cell death or survival. A recent study demonstrated the involvement of eIF2α-dependent pathway of autophagy induction in prostate cancer cells upon PI treatment. This autophagy induction induced a cytoprotective effect in prostate cancer cells which was alleviated when cells were co-treated with autophagy inhibitors [81]. The exact mechanism of autophagy induction followed by PI treatment still remains obscure and needs detailed investigation. Bortezomib treatment induces autophagy in MCF-7 cells as a compensatory mechanism to escape cell death which can be restored upon co-treatment with 3-MA, an autophagy inhibitor [82]. MG-132 induced G2/M arrest and apoptosis was also enhanced upon abolition of autophagy in gastric cancer cells [83]. Similarly, celastrol, a natural PI, resulted in the induction of autophagy and G2/M arrest in HeLa cells. Interestingly it also caused paraptosis, formation of cytoplasmic vacuoles and apoptosis in multiple cancer cell types [84]. On the contrary, MG-132 induced cell death was partially attenuated on treatment with autophagy inhibitor, 3-MA, which ensures the importance of autophagy in PI, triggered cell death. However, induction of autophagy is supposed to be primarily a defense mechanism to bypass the incidence of cell death after PI treatment, still it remains to be a crucial event in PI induced growth arrest of cancer cells (Figure 4). These studies clearly indicate that the intricate mechanisms of association of ER stress, autophagy and resistance to PI therapy needs further investigation.

## Conclusions and future perspectives

**Figure 5:**
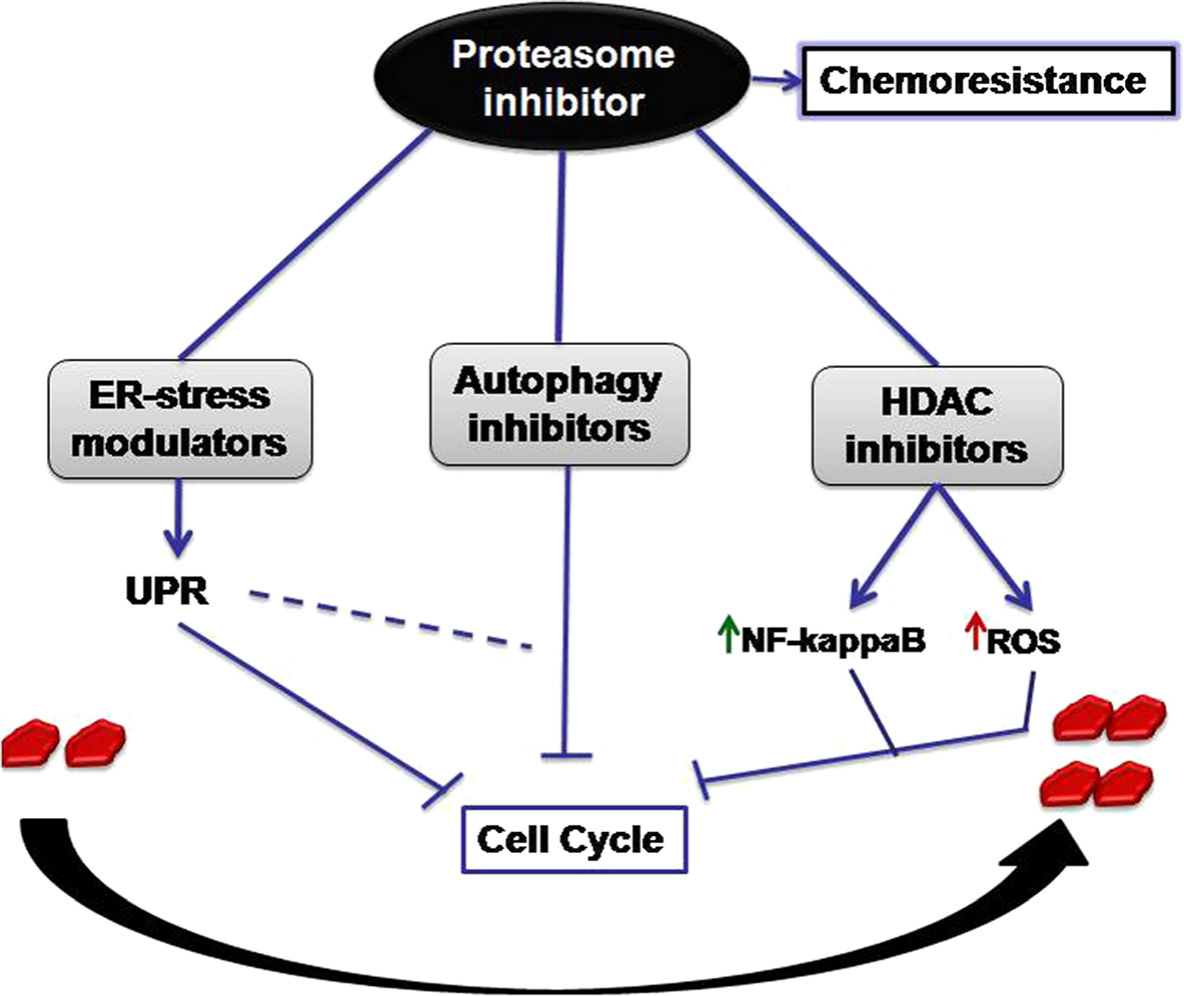
**Refinement in PIs therapy via synergistic approaches.** PIs can be used in combination with different class of compounds for effective targeting of PI resistant cancer cells and overcoming chemoresistance. PIs can be combined with ER-stress modulators which can together cause cell cycle arrest via unfolded protein response and sometimes autophagy. Autophagy inhibitors have also shown to cause cell cycle arrest in combination with PIs thereby surpassing chemoresistance induced by PIs in alone. One more class of compounds, HDAC inhibitors have also shown promising results in combination with PIs. This combination is reported to decrease NFkB activation and induces ROS leading to cell cycle arrest in cancer cells.

Development of chemoresistance against PIs and its inefficacy against many solid cancers necessitates a thorough assessment of the treatment strategies involving PIs. Though not much has been explored in this context but certain reports suggest that induction of autophagy post PI treatment is a major cause of resistance to PI therapy [85-87]. Therefore exploring synergistic drug combinations with PIs for effectively combating chemoresistance and limitations of targeted therapy could be a logical therapeutic strategy. As discussed above proteasome inhibitor induced cell cycle arrest is attributed to modulation of one or more pathways. Therefore, the combination of PIs along with the modulators of one of these signaling pathways, may possibly be explored for their therapeutic potential in PI resistant cancer types. Already some reports have indicated the potential of PIs in combination with different class of compounds (Figure 5). Combination of Bortezomib and autophagy inhibitor, Bafilomycin A1 increases the cytotoxic effects against multiple myeloma cells. Similarly, combination of PIs and ER stress inhibitor, salubrinal potentiates toxicity in therapy resistant multiple myeloma cells [88,89]. Recent studies also suggest the synergy between HDAC inhibitors (HDACi) and PIs against multiple myeloma cell lines and have shown promising results in clinical trials [89]. PIs in combination with HDAC inhibitors also induce cell cycle arrest via NFkB and ROS pathways [90]. These findings provide new insights for combination therapy of HDAC inhibitors and PIs [91,92]. Bortezomib has also been tested in combination with Nelfinavir, an HIV protease inhibitor and the results have indicated induction of ER stress, cell cycle arrest in cervical cancer cells [93]. Several other combinations with PIs worked out on different cancer cells given encouraging results for combination therapy [94-100] is summarized in Additional file 1: Table S1. However, there is an ever increasing need for such synergistic combinations with drugs from the synthetic and natural source for effectively targeting cancer cells with PI resistance.

## Abbreviation

PI: Proteasome inhibitor; CDK: Cyclin dependent kinase; CKI: Cyclin dependent kinase inhibitor; UPP: Ubiquitin proteasome pathway; UPR: Unfolded protein response; ER stress: Endoplasmic reticulum stress; 3-MA: 3-Methyladenine; NF-ĸB: Nuclear factor kappa B; SCF: Skp 1/Cul 1/F-box protein; APC: Anaphase promoting complex; UBE2C: Ubiquitin-conjugating enzyme E2C; ROS: Reactive oxygen species.; JNK: c-jun-N-terminal kinase; MAPK: Mitogen activated protein kinase; DDR: DNA damage response; HDACi: Histone deacetylase inhibitors.

## Competing interest

The authors declare that there is no competing interest.

## Authors’ contribution

NR collected and reviewed the literature and wrote the manuscript. DPM corrected and revised the manuscript. Both authors read and approved the final manuscript.

## Authors’ information

The research group headed by DPM is dedicated to study of molecular mechanisms of cell death in cancer, neuronal and male germ cells. We are currently screening for proteasome inhibitors using commercially available drug libraries and natural compound libraries synthesized at the CSIR- Central Drug Research Institute, Lucknow, India.

## Supplementary Material

Additional file 1 **Table S1.** List of combinations of PIs with different class of compounds targeting cancer cell division. Click here for file
