# Supplementary material for: Therapeutic targeting of cancer cell cycle using proteasome inhibitors
Source: Cell Div. 2012 Dec 26;7:26. doi: 10.1186/1747-1028-7-26 (PMC3584802; doi:10.1186/1747-1028-7-26)
Supplement: Additional file 1 — Table S1. List of combinations of PIs with different class of compounds targeting cancer cell division. [file 1747-1028-7-26-S1.doc]

**Supplementary Table 1 List of combinations of PIs with different class of compounds targeting cancer cell division**.

| **PIs in Combination** | **Cancer type** |
| --- | --- |
| Salubrinal and Bortezomib/MG-132^89^ | Multiple myeloma |
| PCI-24781 and Bortezomib^92^ | Lymphoma cells |
| Romidepsin/Belinostat and Bortezomib^95^ | Mantle cell lymphoma |
| Valproic acid and Bortezomib^96^ | EBV positive and negative T and NK lymphoma cells |
| Nutlin-3 (mdm2 antagonist) and bortezomib^97^ | Mantle cell lymphoma |
| P276-00 (CDK inhibitor) and Bortezomib^98^ | Multiple myeloma |
| PD 0332991 and Bortezomib^99^ | Multiple myeloma |
| MS 275 and Bortezomib^94^ | Cholangiocarcinoma cells. |
| CDK4/ CDK6 inhibitor and Bortezomib^100^ | Multiple myeloma |
